# Lipid (per) oxidation in mitochondria: an emerging target in the ageing process?

**DOI:** 10.1007/s10522-017-9710-z

**Published:** 2017-05-24

**Authors:** O. S. Ademowo, H. K. I. Dias, D. G. A. Burton, H. R. Griffiths

**Affiliations:** 1 Life & Health Sciences, Aston University, Birmingham, UK; 2 Health and Medical Sciences, University of Surrey, Guildford, UK

**Keywords:** Fatty acid, Oxidised phospholipid, Ageing, Membrane lipid remodelling, Mitochondria, Antioxidant, Cellular senescence

## Abstract

Lipids are essential for physiological processes such as maintaining membrane integrity, providing a source of energy and acting as signalling molecules to control processes including cell proliferation, metabolism, inflammation and apoptosis. Disruption of lipid homeostasis can promote pathological changes that contribute towards biological ageing and age-related diseases. Several age-related diseases have been associated with altered lipid metabolism and an elevation in highly damaging lipid peroxidation products; the latter has been ascribed, at least in part, to mitochondrial dysfunction and elevated ROS formation. In addition, senescent cells, which are known to contribute significantly to age-related pathologies, are also associated with impaired mitochondrial function and changes in lipid metabolism. Therapeutic targeting of dysfunctional mitochondrial and pathological lipid metabolism is an emerging strategy for alleviating their negative impact during ageing and the progression to age-related diseases. Such therapies could include the use of drugs that prevent mitochondrial uncoupling, inhibit inflammatory lipid synthesis, modulate lipid transport or storage, reduce mitochondrial oxidative stress and eliminate senescent cells from tissues. In this review, we provide an overview of lipid structure and function, with emphasis on mitochondrial lipids and their potential for therapeutic targeting during ageing and age-related disease.

## Introduction

Ageing can be considered as a progressive failure in the maintenance of tissue homeostasis leading to impaired function, health decline and the emergence of age-related disease. Ageing likely involves the dysregulation of several interconnected biological processes including redox state, metabolism, cell signalling, repair, replicative and adaptive capacity (Calabrese et al. 2010). Tightly regulated metabolism, the synthesis and degradation of macromolecules, provides a fundamental quality control check and energy resource. Recent evidence is emerging to indicate that in addition to the longstanding link between impaired lipid metabolism and atherosclerosis, lipid metabolism is also related to ageing. However, while altered lipid metabolism has been associated with and related to age-related diseases (Tindale et al. 2017; Dmitriev and Titov 2010; van Diepen et al. 2013), it is often difficult to discern whether such changes are a cause or a consequence of ageing.

Age-associated impairment in mitochondrial function, including dysregulation of lipid metabolism and increased reactive oxygen species (ROS) production, may be one mechanism contributing to changes in cellular energetics. The majority of mitochondrial lipids are synthesised in the endoplasmic reticulum (ER) and transported to the mitochondria, but some lipids (cardiolipin, phosphatidylethanolamine) are known to be synthesised within the inner membrane of the mitochondria and are critical for maintaining the unique architecture of the mitochondrial membranes, the cristae (Schlame and Greenberg 2017). An increase in ROS production may further exacerbate mitochondrial dysfunction, partly via oxidative damage to lipids (peroxidation), oxidation of respiratory chain proteins affecting metabolism and protein import, and in inducing DNA damage. The age-related accumulation of mitochondria DNA (mtDNA) mutations and respiratory chain dysfunction may lead to a decline in mitochondrial function associated with increased mitochondrial ROS production and a decline in ROS-scavenging enzymes (Simoncini et al. 2015). Mechanistic links between lipid metabolism and inflammation have also been established, where lipids can directly activate inflammatory pathways and vice versa (Pararasa et al. 2016; van Diepen et al. 2013). In addition, mitochondrial dysfunction has been linked to a shift in cellular metabolism towards glycolysis, highlighting the tight relationship between metabolism, energetics and ageing (Selfridge et al. 2013).

If indeed mitochondrial dysfunction contributes to significant lipid peroxidation, impaired lipid biosynthesis and ageing itself, then therapeutic stabilisation and restoration of mitochondrial function via targeted treatment with antioxidants (Zhang et al. 2016; Cai et al. 2015) may prove beneficial for alleviating age-related decline.

## Lipid metabolism and lipid composition changes with ageing

Lipids are essential and multifunctional; they are structural components of cell membranes, provide energy reserves, mediate signal transduction and indirectly, can regulate gene expression (Mecocci et al. 1996; Elustondo et al. 2017). Interactions with specific receptors or enzymes mediate the biological effects of many lipid classes including ceramides, eicosanoids, phosphoinositides, cholesterol and sphingolipids. In turn, lipids play important roles in the regulation of cell proliferation, apoptosis, inflammation, migration and adhesion (Ng et al. 2017; Helkin et al. 2016; Mollinedo and Gajate 2015; Greig et al. 2012). Lipids, especially phospholipids (PL) have important mitochondrial functions due to their prominent roles in cell membrane formation, cell signalling pathways and energy storage. Free fatty acids (FFAs) are the major lipid constituents of membrane phospholipids and during disease conditions phospholipases are activated inducing PL hydrolysis and the release of FFAs. Phosphatidylcholine (PC) is the most abundant phospholipid present in the outer layer of the cellular membrane and its concentration typically increases with age (Hong et al. 2016). In Alzheimer’s disease (AD), Hong et al. recorded a decrease in PCs and increase in lysophatidylcholine (LPC) formed by hyperactivated phospholipase 2 (Hong et al. 2016). PL enable important neuronal functions including neurotransmission, stabilisation of ion channels and the localisation of β-amyloid plaques to PL cores (Hong et al. 2016).

Many age-related diseases including cardiovascular diseases, diabetes, kidney disease, rheumatoid arthritis and neurodegenerative diseases are associated with altered lipid metabolism and consequently changes in lipid profiles (Greig et al. 2012; AbouAssi et al. 2017; Zhang and Forman 2016; Helkin et al. 2016; Pararasa et al. 2015). Whiley et al. identified that some long chain PCs (16:0/20:5; 16:0/22:6 and 18:0/22:6) were at significantly lower concentrations in AD patients, particularly 16:0/20:5 (Whiley et al. 2014); noticeable reduction of sulfatides has also been found in AD (Hong et al. 2016). Gaudin et al. also found that PC with very long chain fatty acids (32:0 and 34:0) increased in AD (Gaudin et al. 2012). Conversely, sphingomyelin is reported to be at lower concentrations in AD patients compared to controls (Hong et al. 2016; Whiley et al. 2014).

Dysregulation in lipid desaturation may disrupt normal cell processes, for example, the decline in omega 3 fatty acids that is observed in ageing is considered proinflammatory. This is likely to arise from lower expression of the delta 6 desaturase enzyme. An inter-relationship between mitochondrial gene expression and lipid profiles was demonstrated in an elegant GWAS study; ten genetic variants-mtSNPs were associated with HDL cholesterol and triglyceride levels, highlighting the importance of the mitochondrial genome to the regulation of lipid levels (Flaquer Rospleszcz et al. 2015) in cardiovascular disease.

One of the most visible signs of ageing is an alteration in visceral and subcutaneous fat deposition; there is less subcutaneous fat deposited under the skin which not only impairs thermostasis but also reduces the potential for glucose and fatty acid disposal and insulin resistance (Torres-Villalobos et al. 2016), probably due to changes in hormone secretion (Pararasa et al. 2015). Fat accumulates at ectopic sites with age, such as the bone marrow, muscle and the liver. This may be cytotoxic to emergent immune cells, satellite cells and increase non-alcoholic fatty liver disease (Heilbronn et al. 2004) and can exacerbate insulin resistance (Gao et al. 2009). The most important fatty acid in lipotoxicity is palmitate that is converted to ceramides intracellularly (Gao et al. 2012). Palmitate, probably via ceramide, is an uncoupler of mitochondria, promoting release of ROS in a range of cell types (Phillips et al. 2002).

Deposition of visceral and subcutaneous fat is dependent on the size of the adipocyte pool and efficiency of converting fatty acids into triglyceride. The peroxisome proliferator activated receptor γ (PPARγ) transcription factor family regulates the expression of genes that catalyse the conversion of fatty acids into triglyceride for storage in a tissue specific manner (Nakamura et al. 2014). Metabolic switching between energy sources can also be controlled by PPARγ in immune cells (Griffiths et al. 2017) highlighting a tight relationship between metabolism and immune function (Torrao et al. 2014). Intriguingly, PPARγ expression by Treg cells is necessary to restore insulin sensitivity in obese mice (Cipolletta et al. 2012). In common with adipocytes, PPARγ is phosphorylated at Ser273 by Cdk5 in visceral adipose tissue Treg cells (Cipolletta et al. 2015).

The importance of fatty acid storage for longevity was elegantly demonstrated in a PPARγ deficient mouse that showed reduced lifespan; moreover, an in silico study suggested that the PPARγ signalling pathway was part of a longevity network (Argmann et al. 2009). With age, there is a decline in pre-adipocyte proliferation and differentiation (Sepe et al. 2011). This is likely to contribute to the increased systemic load of free fatty acids that we and others have described during healthy ageing (Pararasa et al. 2016).

In our previous study of fatty acids in plasma, we showed that the longer chain fatty acids (C14–C22) were increased in the healthy over 65 years of age group. This effect may be related to the observed increase in methylation of the Elongation of Very Long Chain Fatty Acids 2 (ELOVL2) promoter that has been reported in monocytes and T cells with age (Reynolds et al. 2014). Decreased expression of ELOVL2, which catalyses the extension of C20 and C22 poly unsaturated fatty acid (PUFA) (arachidonic acid and eicosopentanoic acid) to form very long chain unsaturated fatty acids, offers an explanation for the accumulation of ELOVL2 substrates that we observed. Both n−6 and n−3 PUFAs are a component of membrane phospholipid thus changes in their frequency will influence both the structure and function of the membrane (Ma et al. 2004). Finally, ELOVL2 also plays an important role in triglyceride storage and lower expression promotes an increase in fatty acid release by adipocytes (Kobayashi et al. 2007).

Consistent with other changes seen in fatty acid metabolism, plasma cholesterol levels tend to increase with age. However, this effect is not uniform across lipid classes; plasma LDL increases whereas HDL concentrations tend to be lower (Davidson et al. 2002; Walter 2009). The net effect is that cholesterol transport into cells is increased but reverse cholesterol transport out of cells to HDL decreases. Cholesterol together with ceramides can confer changes in membrane fluidity in the phospholipid bilayer and affect the formation of lipid raft microdomains; accumulating data suggest that the function of lipid microdomains is altered with ageing and can impair immune signalling (Fulop et al. 2012).

Several studies have highlighted differences in the lipid profile of male and females relating to age. Higher total cholesterol (TC), low-density lipoprotein cholesterol (LDL), and non-high-density lipoprotein cholesterol (N-HDL) in the non-elderly compared with the elderly for both males and females (Wei et al. 2014). Compared with females, males have higher HDL while females have high LDL with no difference in HDL among various age groups for females but there was significance for males (Goh et al. 2007; Wei et al. 2014). Triacylglycerol (TG) was reported to be significantly different in males of different age groups but no difference was observed in TG for females. TG levels were higher in 40–49, 50–59, and 60–69 year age groups in males than those in 70–79, 80–89, and ≥90 year age groups (Wei et al. 2014).

Goh et al. (2007) evaluated the impact of age and gender on lipid and lipoprotein profiles in a cohort of healthy Chinese-Singaporean and found no significant difference in the lipid and lipoprotein levels in men of different age groups but observed significantly higher level of some lipids in older women (>50 years old) than in younger women (30–46 years old). They also reported TG to be significantly correlated with lipids and lipoproteins differently in men and women (Goh et al. 2007).

Reynolds et al. (2010) evaluated the relationship between serum lipids and cognitive decline in men and women from the ages of 50–96 years. Among their findings, TG predicted memory ability in women across all ages with low TG predicting general cognitive ability while in men apolipoprotein B and TC levels correlated with better cognitive function. The gender effect seen in the lipid profile has been associated with the hormone androgen (Reynolds et al. 2010).

Metabolism is required to provide ATP for cell survival. The most efficient mode of ATP production is via oxidative phosphorylation in the mitochondrion and is supplied from the degradation of carbohydrates, proteins and lipids. Normally, oxidative phosphorylation is a tightly coupled process of two electron transfer in the presence of oxygen, yielding ATP and water. However, in conditions of nutrient excess or when components of the electron transport chain are modified, single electrons may leak (Jackson et al. 2002). The first product of single electron leakage is the reactive oxygen species (ROS), superoxide anion. Superoxide itself has relatively low reactivity and probably does not contribute to any major oxidative damage. However, superoxide spontaneously dismutates to hydrogen peroxide which is membrane permeable and is a powerful oxidant generating hydroxyl radicals via the Fenton reaction in the presence of catalytic iron. During oxidative stress, the imbalance in oxidants and antioxidant levels results in oxidative damage to macromolecules (Simoncini et al. 2015; Gandhi and Abramov 2012). The uncontrolled oxidation of lipids mediated by reactive oxygen species (ROS) is known as peroxidation. Peroxidation causes fundamental changes in molecular properties. For example phospholipids are essential for membrane architecture, but peroxidation causes changes in fluidity which can influence receptor clustering and yield bioactive and inflammatory oxidised phospholipids. Peroxidation is considered in the next section.

Hydrogen peroxide can mediate oxidation of thiolate anions found in cysteine residues that have a pKa ~5 and this is considered to be an important reaction for inactivation of tyrosine phosphatases and for propagating inflammatory signalling (Ratnayake et al. 2013). The intermediate sulphenic acid can be reduced by glutathione (GSH), catalysed by glutathione reductase, although this reaction is limited during ageing as GSH concentrations tend to be lower. Superoxide may also combine with nitric oxide to form peroxynitrite, another lipophilic species that can cross biological membranes and exert oxidising and nitrating reactions. Oxidation of cysteine residues in the iron-sulphur clusters of the mitochondria can further exacerbate ROS leakage (Sheeran and Pepe 2016).

During ageing, the integrity of the mitochondrial respiratory chain is compromised. ROS-induced mitochondria DNA mutation/damage, lipid and protein oxidation, affect physiological functions (Cui et al. 2012). In this state, cellular antioxidant defences are insufficient to keep the ROS levels below a toxic level (Simoncini et al. 2015). Age related accumulation of ROS has been suggested to play a part in the damage to major cell components in age-related diseases (Simoncini et al. 2015).

## ROS induced lipid peroxidation

Lipid peroxidation is a biologically important process that results in intermediate unstable oxidised lipid species and stable end products, which act as bioactive lipid mediators. Being more polar than their parent lipids, lipid peroxides (LPOs) can perturb membrane bilayers, affect membrane structure and interfere with intracellular functions. Phospholipid peroxidation changes membrane biophysical properties, decreasing fluidity, inactivating membrane-bound proteins and ultimately leads to destruction of the membrane (Wong-ekkabut et al. 2007).

Products of peroxidation, lipid-oxides and –peroxides, are reported to be involved in many cellular processes including cellular metabolism, signalling, and cell survival (Aufschnaiter et al. 2017). Lipid molecules, especially PUFA and cholesterol, undergo oxidation at varied rates initiated by ROS [e.g. hydroxyl radicals (OH·), superoxide (O_2_·–), peroxyl radical (ROO–), nitric oxide (NO·), peroxynitrite (ONOO·–) or nitrogen dioxide (NO_2_·)], and in PUFA this initiates a self-propagating chain reaction (Halliwell 2001). The propagation stage of lipid peroxidation involves electron transfer and oxidation of other PUFA in close proximity. Propagation and termination have been reviewed extensively elsewhere (Yin et al. 2011; Girotti 1998). Non-enzymatically generated lipid peroxides often have a very short half-life and therefore pose technical challenges for precise measurement and biological study. Mass spectrometry is seen as the method of choice for absolute determination of lipid peroxidation.

In our recent work, we explained some challenges with oxidative lipidomic techniques and reported the detection of lipid oxidation by analysing the novel oxidised phospholipid biomarker 1-palmitoyl-2-(5’-oxo-valeroyl)-sn-glycero-3-phosphocholine (POVPC), a truncated oxidation product of 1-palmitoyl-2-arachidonoyl-sn-phosphatidylcholine (PAPC) using electrospray ionization tandem mass spectrometry (MS) with multiple reaction monitoring (MRM) (Ademowo et al. 2017). Several authors have used antioxidant activities and oxidative stress derived biomarkers to detect and analyse lipid peroxidation (Cai et al. 2017; Umeno et al. 2017; Ademowo et al. 2017). Gaschler and Stockwell reviewed in details several other methods for the detection and analysis of lipid peroxidation in biological samples [see (Gaschler and Stockwell 2017)].

Oxidised phospholipids (oxPLs) are generated from (poly)unsaturated diacyl- and alk(en)ylacyl glycerophospholipids. OxPLs exert a wide variety of biological effects and play a role in the development of several chronic diseases (Philippova et al. 2014). In pathological conditions associated with activation of oxidative stress, unsaturated phosphatidylcholines, the most abundant phospholipids undergo oxidation leading to generation of fragmented phospholipids such as 1-palmitoyl-2-hydroxy-sn-glycero-3-phosphocholine (lysoPC), or 1-palmitoyl-2-arachidonoyl-sn-glycero-3-phosphocholine (PAPC) full length oxygenation products (oxPAPC) (Heffern et al. 2013). Other oxidised derivative of PAPC are 1–palmitoyl-2–(5–oxovaleroyl)- sn -glycero-3–phosphocholine (POVPC), 5–hydroxy-8–oxo-6–octenoyl-phosphocholine (HOOA-PC), 1–palmitoyl-2–(5,6–epoxyisoprostane E2)- sn -glycero-3–phosphocholine (PEIPC), and 1–palmitoyl-2–glutaroyl- sn -glycero-3–phosphocholine (PGPC) (Philippova et al. 2014). Eicosanoids including prostaglandins, thromboxanes and leukotrienes are also products of arachidonic acid oxidation and they play major regulatory roles in immune and inflammatory functions (Rubio-Perez and Morillas-Ruiz 2012).

Although oxPLs are recognized to be involved in disease pathogenesis, others reported that the converse is true under certain biological conditions (Mauerhofer et al. 2016). Mauerhofer et al. have demonstrated that OxPLs can also induce protective effects such as inhibition of inflammatory signaling pathways through Nrf2-dependent and -independent mechanisms, upregulation of antioxidant genes, antagonism of Toll-like receptors. The immuno-modulating and immuno-suppressive action of OxPLs may influence adaptive immunity and autoimmune disease through activation of PPARs which are known for their anti-inflammatory action. OxPLs may also inhibit inflammation induced via TLR4 (Bochkov et al. 2010). Hence, phospholipid oxidation may provide a negative feedback to prevent damage to host tissues from uncontrolled inflammation and oxidative stress (Mauerhofer et al. 2016).

Whilst cholesterol-derived hydroperoxides are more stable and have been studied both in model systems and cells (van Lier et al. 2015), the analysis of the predominant lipid peroxide species in fluids and tissue in health and how they change in disease requires methodological precision and sensitivity (Reis 2017). Due to the instability of many other intermediate products, PUFA-derived stable aldehydes such as 4-hydroxy-2-nonenal (HNE), malondialdehyde (MDA) and acrolein are the most extensive species that are studied (Esterbauer et al. 1991; Ayala et al. 2014; Zhang and Forman 2016). HNE and MDA show high chemical reactivity and interact with proteins, resulting in the formation of covalent adducts that can be measured by ELISA and often modulating protein activity (Liu et al. 2014b) including in proteins in the membrane (Pizzimenti et al. 2013). The majority of studies have focussed on the aforementioned three stable aldehydes, however, advances in analytical capability using advanced MS methods are revealing many other interesting and less well-studied carbonyl compounds (Sousa et al. 2017). Products of lipid peroxidation are often reactive themselves and are classified as either hydroxyl acids or reactive aldehydes (Gaschler and Stockwell 2017). Due to the ability of lipid peroxidation products such as aldehydes to covalently modify proteins, Shibati et al. used mass spectrometry methods to detect product ions generated from positively ionised adducts (Shibata et al. 2017). In future, in depth studies using state of the art MS methods will help us to understand more about targets and potentially mechanisms of lipid peroxidation involving protein adducts in ageing.

Zheng et al. (2005) measured HNE-adduct accumulation in ageing in fruit flies using an ELISA method and found that HNE adduct concentrations are significantly increased in the second half of the adult life by two fold (Zheng et al. 2005). Another study using ageing Wistar rats at 7, 15, 22 and 30 weeks found significantly increased levels of HNE-protein adducts with ageing (Asselin et al. 2006).

Isoprostanes (IsoPs) are prostaglandin F2-like compounds and neuroprostanes (NPs) that are formed by the non-enzymatic, free radical-mediated oxidation of arachidonate and docosahexaenoate, respectively. IsoPs have been measured in biological fluids such as urine, plasma, bile, cerebrospinal fluids, and in many cell and tissue types (Dias et al. 2015; Cracowski 2004; Ademowo et al. 2017). Choksi et al. (2007) showed lower serum and liver IsoP levels at all ages in Dwarf mice that show inherently low levels of ROS. The authors suggested that lower levels of endogenous ROS production may be a factor in their resistance to oxidative stress and longevity (Choksi et al. 2007).

García-Flores et al. (2017) analysed F_2_-dihomo-isoprostanes, F_3_-neuroprostanes, and F_4_-neuroprostanes- in urine samples from 158 healthy volunteers ranging from 4 to 88 years old. They found a significant, positive correlation between age and total F_2_-dihomo-IsoP concentration (García-Flores et al. 2017). To further investigate any relationship between lipid peroxidation and ageing, a cohort study was undertaken in a geriatric population; circulating MDA was related to frailty and not to age or sex (Inglés et al. 2014).

An increase of LPOs is commonly found in many age-related diseases. Skewing of the redox balance towards oxidation is observed in biological ageing and in many inflammatory diseases that increase with age. Table 1 provides examples of organs and diseases that are associated with increased LPOs.

**Table 1:** Increased lipid peroxidation products in age related diseases

Organ	Diseases	Example references
Brain	Trauma, stroke, neurodegenerative diseases	Naudí et al. (2017), Zhang and Forman (2016) and Hall et al. (2016)
Eye	Cataractogenesis, retinal damage	Liu et al. (2014a) and Mark and Anne (2009)
Blood and blood vessels	Atherosclerosis	Dias et al. (2015) and Geng et al. (2015)
Skin	Dermatitis	Zheng et al. (2014)
Heart	Cardiovascular disease	Pytel et al. (2016) and Tejovathi et al. (2013)
Teeth and gum	Periodontitis	Baltacıoğlu et al. (2014) and Akalιn et al. (2007)
Liver	Chronic liver disease	Morita et al. (2012) and Vuppalanchi et al. (2011)
Pancreas	Diabetes, chronic pancreatitis	Wensaas et al. (2009) and Santini et al. (2003)
Lung	Asthma, hypoxia	Wood et al. (2003) and Diamond et al. (2016)
Kidney	Chronic kidney disease	De Vecchi et al. (2009) and Vaziri and Norris (2011)
Bone and joints	Arthritis	Sarban et al. (2005)
Multiple organs	Cancer	Herrera et al. (2014)

To minimise lipid peroxide and reactive aldehyde accumulation to subtoxic levels, cells have a vast network of antioxidant and detoxification systems. Removal of aldehydes is achieved by glutathione (GSH) that reacts with aldehydes to form GSH adducts either spontaneously or catalysed by glutathione S-transferases (GSTs), by aldo–keto reductases (AKRs) to alcohols or oxidation to acids by aldehyde dehydrogenases. The redoxins (thioredoxin, peroxiredoxin, glutaredoxin) and dismutase enzymes also play a role in the detoxification process and have been reviewed elsewhere (Zimniak 2008; Krag 2011). Increased LPO products observed in biological ageing are likely to be a result of increased production and decreased removal caused by age-associated dysfunction of detoxification.

## Mitochondrial lipids in biological ageing and age-related diseases

Mitochondria are cytoplasmic organelles with a characteristic double-membrane structure and have been described as the “powerhouses” of the cell, generating the energy needed to function and survive (Reddy and Beal 2005; Yin et al. 2015; Simoncini et al. 2015). Mitochondria perform electron transport producing adenosine triphosphate (ATP) (Swerdlow and Khan 2004) and regulate the initiation of apoptosis by releasing proteins that activate the caspase family proteases. Disruption of the electron transport chain (ETC) affects the reduction–oxidation (redox) potential of the cell. This impacts on signalling pathways through modulation of SIRT (Griffiths et al. 2017) and by oxidising reduced NADH to NAD^+^ needed in glycolysis (Selfridge et al. 2013). Mitochondria are the greatest cellular source of ROS as the electron transport consumes about 85% of the oxygen that the cell uses (Reddy and Beal 2005). An impaired ETC reduces ATP energy but increases ROS production. During biological ageing, aberrations in the control of cell death, impaired metabolism and perturbed redox homeostasis have been attributed to mitochondrial dysfunction (Yin et al. 2015) and in the pathogenesis of inflammation (Wan et al. 2014) and age related diseases including neurodegenerative diseases (Di Domenico et al. 2016), diabetes (Vadvalkar et al. 2017) and cardiovascular diseases (Li et al. 2017).

As a source of ROS production, mitochondria are particularly susceptible to locally mediated oxidative damage to lipids, DNA and proteins; the latter are normally limited by effective oxidative DNA damage repair enzymes and the Lon protease respectively. Secondary, oxidative DNA damage from lipid peroxidation products may contribute to the increasing numbers of mitochondrial mutations seen in diseases and inactive respiratory complexes, so promoting further ROS leakage in a vicious cycle of oxidative damage (Yao and Brinton 2011; Tanaka et al. 1996).

The structural and functional arrangement of mitochondrial protein complexes within the membrane changes with age. These structural changes affect the ion mobility disrupting the carrier proteins that shuttle ions, ATP, ADP and small metabolites between the cytoplasm and the matrix; these proteins include the 33 kDa ATP/ADP carrier, the soluble electron carrier protein, cytochrome c and enzymes such as the ATP synthase, succinate dehydrogenase and other oxido-reductases (Pebay-Peyroula et al. 2003; Kühlbrandt 2015). However, whether or not the morphological changes seen in mitochondria during ageing are direct or indirect consequences of oxidative damage to proteins and lipids is not known. The biogenesis of mitochondrial proteins during ageing is not well explored and due to the dynamic and fragile nature of mitochondria protein complexes, they are not easily analysed by established methods (Kühlbrandt 2015). Hence the effects of age-related changes to mitochondrial lipid and protein complexes are emerging areas of research.

Cardiolipin (CL), a form of glycerophospholipid found in the inner membrane of the mitochondrial lipid bilayer, has an important structural role acting as an anchor for respiratory super-complexes and for mitochondrial DNA during replication. CL is essential for mitochondrial health (Shen et al. 2015) and CL oxidation has been identified as a primary event in the release of cytochrome c and to increase permeability of the mitochondrial membrane to apoptosis factors such as BAX and BAD. The release of cytochrome c was suppressed when the production of ox-CL in mitochondria was inhibited by the overexpression of mitochondrial phospholipid hydroperoxide glutathione peroxidase (Nomura et al. 2000).

As an anionic phospholipid, cardiolipin is very susceptible to oxidation; this affects respiration, the assembly and stability of the mitochondrial protein import machinery, may cause abnormal mitochondrial morphology and even promote cell death (Horvath and Daum 2013). Recently, a pentapaptide-SS31 has been discovered to bind and protect cardiolipin from oxidation. It has been shown to improve mitochondrial function in ageing muscle, maintain the structure of cristae and protect neurons from hypoxia reperfusion injury (Birk et al. 2014).

### Mitochondrial lipids in neurodegeneration

Although the central nervous system (CNS) function is dependent on efficient mitochondrial activity due to its energy requirement, it is still unclear whether or not mitochondrial impairment and oxidative stress are actually involved at the onset or progression of neurodegeneration and some other diseases (Simoncini et al. 2015). Mitochondria are rapidly transported to areas of high energy demand such as the neuronal projections (Selfridge et al. 2013). The mitochondrial transport is important for the development, function and stability of synapses and dendritic spines (Selfridge et al. 2013).

Mitochondrial dysfunction has been reported to be one of the earliest and prominent features in neurodegenerative disorders as ROS are produced locally and target the mitochondria causing the oxidation (Simoncini et al. 2015). Emerging research has strongly implicated mitochondrial dysfunction as causal in the brain energy deficit and failure to adapt to stressors observed in neurodegenerative diseases such as AD; mitochondrial abnormalities are part of AD phenotype (Selfridge et al. 2013). The decline in glucose metabolism and mitochondrial function with increased oxidative stress often appears decades before onset of clinical/pathological symptoms of the disease (Yao and Brinton 2011), hence, ROS have been suggested to be the cause rather than the consequence of neurodegeneration (Moreira et al. 2009). During AD progression, lower glucose utilization and exhaustion of the ketone reservoir increases mitochondrial dysfunction and the fatty acid oxidation pathway which results in white matter degeneration and eventually neuronal death (Yao and Brinton 2011).

Fundamental changes in the composition and distribution of lipids within the brain are believed to contribute to the cognitive decline associated with ageing. The mechanisms by which these changes in lipid composition affect cellular function and ultimately cognition are not well understood (Kennedy et al. 2016; Hong et al. 2016; Whiley et al. 2014). However, Kennedy et al. identified the mitochondria as an important downstream target of PC(O-16:0/2:0), a neurotoxic lipid species found to be increased in AD and that PC(O-16:0/2:0) promotes a global increase in ceramide accumulation in human neurons, associated with mitochondrial-derived ROS and toxicity. These data suggest that PC(O-16:0/2:0)-dependent mitochondrial dysfunction may be an underlying factor for increased ROS production associated with AD (Kennedy et al. 2016). The mitochondrion-located antioxidant enzyme copper/zinc superoxide dismutase is significantly decreased in the frontal and temporal cortex of AD brain, rendering the AD mitochondrion at increased risk for oxidation and offering a possible explanation as to why brain cells (astrocytes, neurones and glial cells) are more susceptible to mitochondrial dysfunction in AD (Reddy and Beal 2005; Moreira et al. 2009).

### Mitochondrial lipids in diabetes

Type 2 diabetes mellitus (T2DM) is the most common human endocrine disease and is characterized by peripheral insulin resistance and pancreatic islet β-cell failure resulting fat deposition in adipose tissue. Pancreatic islet β-cell mitochondria promote lipid oxidation, increase lipogenesis and activate several stress-response pathways in diabetes (Chattopadhyay et al. 2015).

Kahle et al. (2015) took a targeted metabolomics approach to identify high fat (HF) diet-induced modifications in membrane lipid and mitochondrial-membrane protein signatures in mouse liver cells. After 7, 14, and 21 days exposure to an HF diet rich in polyunsaturated fatty acids, hepatosteatosis was induced and major lipid constituent signatures in liver were modified e.g. increased total unsaturated, long-chain fatty acid-containing acyl-carnitine or membrane-associated diacylglycerol moieties and decreased total short-chain acyl-carnitines, glycerophosphocholines, lysophosphatidylcholines, or sphingolipids. The HF diet progressively decreased the abundance of protein-components of all mitochondrial respiratory chain complexes, inner and outer mitochondrial membrane substrate transporters impacting on energy metabolism in liver cells (Kahle et al. 2015).

Using shotgun lipidomics data with multidimensional mass spectrometry, Han et al. (2005) identified a significant decrease in cardiolipin levels and alterations of cardiolipin acyl chain remodelling in diabetic murine myocardium, linking altered substrate utilization and metabolic flux with the diabetic heart (Han et al. 2005). Transgenic expression of cardiolipin synthase in a streptozotocin-induced diabetic mouse model, accelerated cardiolipin remodelling, improved mitochondrial function and attenuated mitochondrial dysfunction during diabetes (Kiebish et al. 2012). This study highlights an avenue to target mitochondrial bioenergetics via cardiolipin synthase.

### Mitochondrial lipids in cardiovascular diseases

Mitochondria play an important role in cardiomyocytes by sustaining ATP production to satisfy the high demand for energy and the normal function of the heart. Even subtle alterations in mitochondrial function or membrane potential e.g. through peroxidation, can cause a significant change in cardiomyocyte energy production and deleterious consequences in cardiovascular health (Pytel et al. 2016). Increased mitochondrial ROS and reactive nitrogen species production during the early stages of hypoxia are associated with cardiomyocyte toxicity (Kolamunne et al. 2013). Lower levels of CL were observed in the rat ischemia–reperfusion model and a decrease in mitochondrial complex III activity was also observed (Paradies et al. 2004). A proteomic study by (Pavón et al. 2011) revealed that proteins of the electron transport chain constituted the greatest percentage of altered lipid-adducted proteins in rat cardiac mitochondria treated with a spectrum of lipid peroxidation products including HNE and 4-hydroxyhexanal (HHE). While a high level (≥20 µM) of HNE was cardio-toxic, levels around 5 µM of HNE induced endogenous anti-oxidant systems through Nrf2 activation (Dasuri et al. 2009).

As mitochondria have been referred to as the most important determinant of life span (Sun et al. 2014), there is a need to understand mitochondrial bioenergetics in prodromal and extant age-related diseases; to explore signature markers of disease that identify onset and progression; and to investigate different interventions that could prevent or delay the progression of such diseases.

## Mitochondrial ROS production during cellular senescence

As we have discussed previously, impaired mitochondrial function and elevated ROS production associate with altered lipid metabolism and increased lipid oxidation that is observed in several age-related diseases. Senescent cells are one potential source of mitochondrial dysfunction during ageing.

Cellular senescence can be considered as a programmed change in cell state, associated with irreversible proliferative arrest and often accompanied by conversion to a pro-inflammatory, immunogenic phenotype (Burton and Krizhanovsky 2014). In contrast, cell ageing occurs as a result of accumulating stochastic damage over time, leading to gradual impairment in cell function. Senescent cells are not aged/old cells. Although, cell senescence can be induced at random, independent of age, the accompanying changes associated with cell senescence are highly regulated.

Cells become senescent in response to various stress-inducing stimuli such as ROS, telomere dysfunction and oncogene activation, which consequently initiates a persistent DNA damage response (DDR) (Di Micco et al. 2006; Rodier et al. 2009; Rossiello et al. 2014). In recent years, cell senescence has emerged as a major mechanism promoting biological ageing and age-related diseases (Childs et al. 2015; Muñoz-Espín and Serrano 2014; van Deursen 2014) and as such, a discussion on the potential connection between cellular senescence, mitochondrial dysfunction and lipid metabolism is warranted.

The senescent state has been shown to be associated with a shift from mitochondrial respiration towards glycolysis (Aird and Zhang 2014; James et al. 2015) and whilst mitochondrial dysfunction may contribute to this metabolic shift, induction of glycolysis itself could possibly contribute to mitochondrial dysfunction (Senyilmaz and Teleman 2015). An elevation in ROS production in response to impaired mitochondrial function could theoretically activate a DDR, triggering cellular senescence and activating their pro-ageing responses.

To determine whether mitochondrial dysfunction can promote cellular senescence, Wiley et al. depleted mtDNA in IMR90 fibroblasts, treated cells with electron transport chain inhibitors (rotenone or antimycin A) or depleted cells of the mitochondrial chaperone HSPA9 (Wiley et al. 2016). All treatments were shown to induce cell senescence as evident by reduced BrdU incorporation and increased senescence-associated beta galactosidase staining. To evaluate the potential role of mitochondria in orchestrating the pro-ageing features of the senescent phenotype, Correia‐Melo et al. demonstrated that senescent cells lacking mitochondria reduced many aspects of the senescent phenotype including the pro-inflammatory secretome, whilst maintaining ATP production via enhanced glycolysis (Correia-Melo et al. 2016). Findings by Passos et al. suggest that ROS are initially important for the maintenance of cell senescence through sustaining a persistent DNA damage response (DDR) (Passos et al. 2010). This may suggest that ROS production is initially beneficial during the transient appearance of cell senescence, but if senescent cells persist in tissues, likely owing to impaired clearance by the immune system (Burton and Faragher 2015), persistent senescent cells later become harmful.

In principle, these data suggest that disruption to mitochondrial function could promote cellular senescence, in addition to regulating the pro-ageing features of senescent cells. However, these studies do not provide evidence that such mechanistic processes occur during natural ageing in the absence of experimental intervention. Nevertheless, because senescent cells are associated with many age-related diseases, whether mitochondrial dysfunction is a cause or a consequence of cell senescence may not be a primary point of issue. What is of concern is the long-term biological impact of altered processes such as lipid metabolism and lipid peroxidation when senescent cells persist.

## Lipids and lipid peroxidation during cellular senescence

**Fig. 1:**
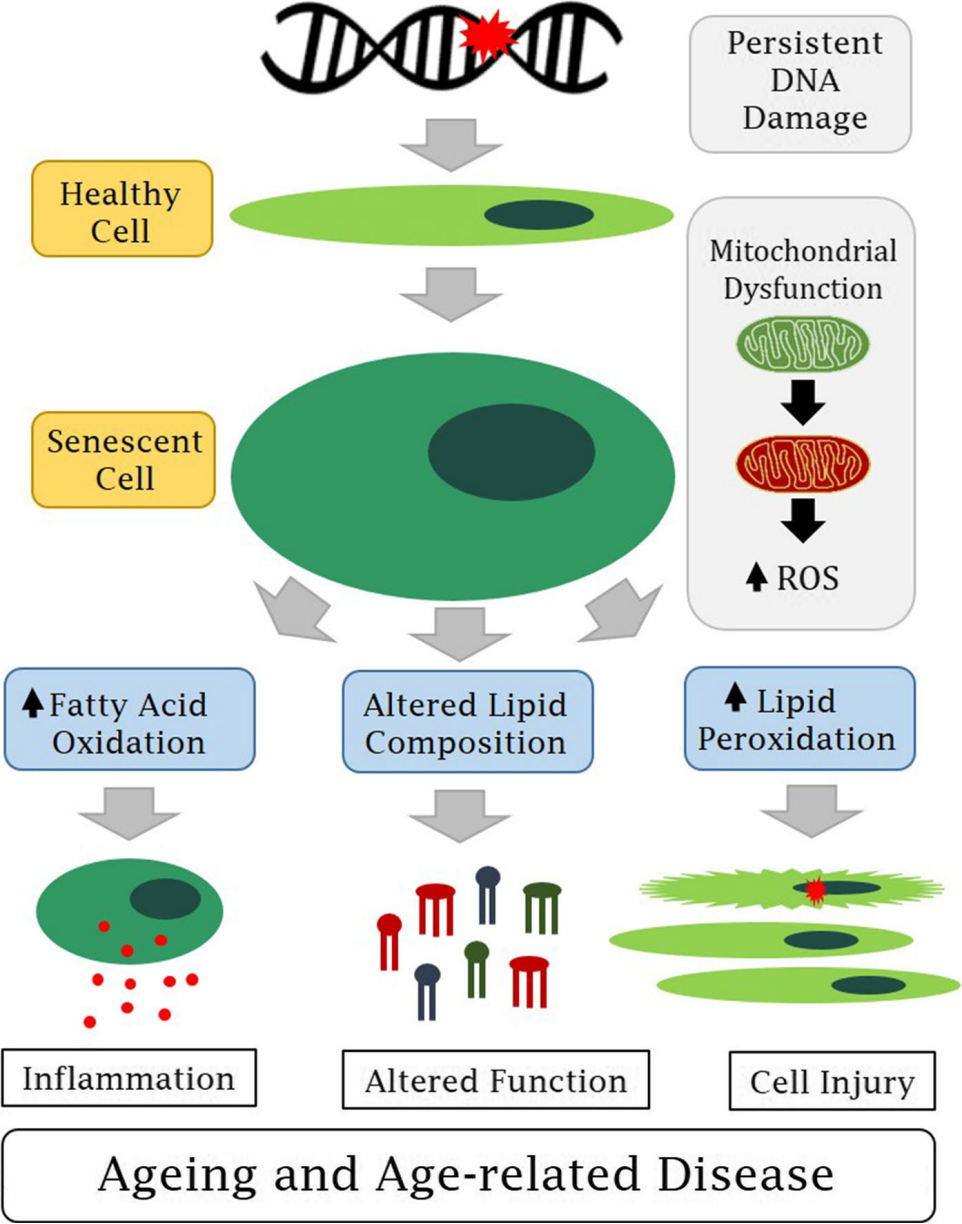
Schematic demonstrating the possible link between cellular senescence, altered lipid metabolism and organismal ageing

Mitochondrial dysfunction, increased lipid peroxidation and altered catabolism will affect the cellular lipidome during cell senescence. Alterations in lipid metabolism and the generation of oxidised lipids may be beneficial for the senescent program during the early stages of senescence induction, possibly through modulating inflammatory and immune responses (Lawrence et al. 2002; van Diepen et al. 2013; Yaqoob 2003). However, if senescent cells persist in tissues, changes in lipid composition can result in cell dysfunction, altered rates of fatty acid oxidation that can induce inflammation and increased lipid peroxidation that can promote damage to neighbouring cells (Fig. 1). These factors may contribute to ageing and age-related diseases. Research focused on altered lipid metabolism during cellular senescence, particularly regarding mitochondrial lipids, is in its infancy. However, in recent years, several studies have made progress in evaluating the senescent lipidome of fibroblasts.

One group investigated the alterations in a number of metabolites associated with the extracellular metabolome of fibroblasts induced to senesce via proliferative exhaustion or via γ-irradiation (James et al. 2015). They reported that a number of fatty acids and their precursors such as eicosapentaenoate, malonate, 7-alpha-hydroxy-3-oxo-4-cholestenoate and 1-stearoylglycerophosphoinositol were elevated during fibroblast senescence when compared with proliferating and quiescent cells, whereas linoleate, dihomo-linoleate, 10-heptadecenoate were depleted. Also amongst the secretory lipidome from senescent fibroblasts was an accumulation of monohydroxy fatty acids (2-hydroxypalmitate, 2-hydroxystearate, 3-hydroxydecanoate, 3-hydroxyoctanoate) and a phospholipid catabolite (glycerophosphorylcholine). It was suggested that whilst some of these changes may be due to oxidative stress, other observed increases may be a response to increased biomass commonly observed amongst senescent cells.

Maeda et al. (2009) investigated the regulation of fatty acid synthesis and ∆9-desaturation during cell senescence in human fibroblasts (Maeda et al. 2009). They found that the levels of fatty acid synthase and stearoyl-CoA desaturase-1 were decreased in senescent fibroblasts compared to proliferating fibroblasts, consequently leading to a decrease in monounsaturated fatty acids. In addition, reduced de novo synthesis of phospholipids with an associated increase in the formation of cholesterol in senescent cells was also observed and exogenous fatty acids were shown to be preferentially incorporated into the triacylglycerol pool of senescent cells.

In another study, the metabolic alterations associated with oncogene-induced senescence (OIS), using Ras-induced senescent human fibroblasts as a model were investigated (Quijano et al. 2012). Through the profiling of ~300 different intracellular metabolites, these authors showed that cells that have undergone OIS develop a metabolic signature which is distinct from cells which have undergone replicative senescence in response to extended in vitro cell culture. In the latter, a switch towards glycolysis has been observed that precedes the onset of senescence (Bittles and Harper 1984). In OIS, an increase in certain intracellular long chain fatty acids, including eicosanoate, dihomo-linoleate, mead acid and docosadienoate were observed. This altered metabolome was shown to associate with a decline in lipid synthesis and increases in fatty acid oxidation. Interestingly, the pro-inflammatory activity of the senescent secretome was reduced by inhibition of carnitine palmitoyltransferase 1, the rate limiting step in mitochondrial fatty acid oxidation, suggesting that alterations in lipid metabolism during OIS may play a role in regulating the pro-inflammatory senescent secretome. Although the mechanism underlying the increase in fatty acid levels during OIS were not fully explored, it may be due to promyelocytic leukemia (PML) activation of the fatty acid oxidation pathway through PPAR signalling (Aird and Zhang 2014). The differences between replicative senescence and OIS are intriguing; they may relate to the physiological need in preventing cancer to switch away from glycolysis as a rapid source of energy that is harnessed by cancer cells to enable them to proliferate rapidly versus the increasing insulin resistance that is seen in ageing and which associates with impaired oxidative metabolism (Burkart et al. 2016). However, while this and other studies have indicated an increase in glucose uptake during OIS, a number of other studies have observed either no change or a significant decrease in glucose uptake. This may relate to the timing of senescence induction, the cell type or the oncogene responsible.

A further study compared global lipid profiles and associated mRNA levels of proliferating and replicative senescent BJ fibroblasts; 19 specific polyunsaturated triacylglycerol species were identified as undergoing significant changes in lipid composition during cell senescence (Lizardo et al. 2017). In addition, significant changes in the expression of genes involved in specific lipid-related pathways, including glycerolipid metabolism, glycerophospholipid metabolism, unsaturated fatty acid synthesis and sphingolipid metabolism were observed during cell senescence. Based on these lipidomic and transcriptomic analysis, the authors postulated that activation of CD36-mediated fatty acid uptake and alteration to glycerolipid biosynthesis may contribute to the accumulation of triacylglycerols during cell senescence. It was suggested that these changes may be a mechanism to prevent lipotoxicity during elevated oxidative stress conditions during cell senescence.

In addition to an altered lipidome during cellular senescence, elevated ROS, likely from uncoupled mitochondria, can promote lipid peroxidation which potentiates cellular damage at distant sites. For example, stable aldehydes can diffuse from their site of generation and form adducts at distant locations, thereby propagating the responses and injury initiated by ROS (Ramana et al. 2013), including the induction of cell senescence in neighbouring cells. Flor and Kron observed an accumulation of lipid-derived aldehydes such as 4-hydroxy-2-nonenal (4-HNE) during accelerated senescence (Flor and Kron 2016). Whereas, the treatment of cells with either 4-HNE or low (5 Gy) γ-irradiation only generated low levels of cell senescence, combining both 4-HNE and 5 Gy γ-irradiation significantly elevated the senescence response. Furthermore, the use of the aldehyde-sequestering drug hydralazine blocked cell senescence induction by 25 Gy and etoposide treatment, demonstrating the potential importance of lipid peroxidation during therapy-induced senescence (Flor et al. 2016). Despite the highly damaging and pro-ageing potential of senescence-derived lipid peroxidation, little research has been conducted in this area and this requires further study.

Research on cell senescence has primarily been undertaken on fibroblasts and more research is required to explore whether the same phenomena are observed in cell-types linked to age-related disease such as in senescent adipocytes, pancreatic beta cells, renal proximal tubular epithelial cells and vascular endothelial cells. Whilst different types of senescent cells may share similarities in lipid metabolism, there may also be differences that are cell type-dependent or due to the mechanism of senescence induction and these require further study to better assess the role of altered lipid metabolism during ageing and disease. Finally, an important question to contemplate is whether the alterations in ROS, lipid metabolism and mitochondrial lipids observed during ageing and diseases are due solely to the presence of senescent cells or whether lipidomic changes can occur in absence of senescent cell accumulation.

## Therapeutic opportunities

Agents that are capable of improving mitochondrial bioenergetics, enhancing glucose metabolism and reducing oxidative stress can promote healthy span (Zhang et al. 2016). Exercise has been found to stimulate mitochondrial biogenesis and activate the phase II antioxidant defence system and improved mitochondrial function is implicated in preventing age-related diseases (Sun et al. 2014). A class of mitochondria-located natural antioxidants are the carotenoids. AD patients exhibit significantly lower serum concentrations of carotenoids such as lutein, lycopene and zeaxanthin associated with increased lipid peroxidation products (Nolan et al. 2014). The enzymes responsible for metabolizing carotenoids are partitioned in the mitochondrial membranes and chronic lycopene administration significantly restores the mitochondrial respiratory enzyme activities in β-A_1-42_ treated rats (Horvath and Daum 2013). Prakash and Kumar studied the neuroprotective effect of lycopene against the β-amyloid induced cognitive impairment and mitochondria oxidative damage in rats. β-A1-42 treated animals showed poor memory retention and marked oxidative stress was indicated by significant increase in oxidative mitochondrial damage, IL-6, TNF-α, and caspase-3 activity. However, the administration of lycopene improved memory retention, attenuated mitochondrial-oxidative damage, reduced neuro-inflammation and restored brain-derived neurotrophic factor levels in β-A1-42 treated rats. The study also indicated that lycopene protected against β-A1-42 induced cognitive dysfunction and modulated amyloidogenesis (Prakash and Kumar 2014). Interventions using targeted carotenoids has the potential to rescue brain mitochondrial lipids, metabolism, redox state and energy stability, thereby increasing lifespan and delaying the appearance of disease (Kelly et al. 2015), but supplementation with these nutrients may only support and protect cognitive health if achieved early enough (Nolan et al. 2015).

Finally, more generally, inhibiting enzyme-catalysed lipid oxidation has also proven beneficial because of the role that oxidised arachidonic acid products, the eicosanoids, play in inflammation that underpins ageing. Non-steroidal anti- inflammatory drugs (NSAIDs), that include acetic acid, salicylate, propionic acid, fenamate, oxicam, and the COX-2 inhibitor classes, have analgesic, antipyretic, and anti-inflammatory properties. They are reported to have a beneficial effect on some neurodegenerative diseases (Rubio-Perez and Morillas-Ruiz 2012).

### Mitochondria-specific antioxidants as a potential therapy in ROS-related diseases

There is a need to explore the potential of mitochondria-specific antioxidants as novel therapeutic strategies targeted towards the prevention, delay or treatment of mitochondria dysfunction in diseases related to mitochondria dysfunction (Mdaki et al. 2016).

An imbalance in the activity of ETC complexes I, II and III leads to ROS generation in both the mitochondrial matrix and cytosol, resulting in glutathione depletion and increased lipid peroxidation. A mitochondrion-targeted antioxidant peptide, SS31, has been reported to protect against oxidative damage (Ma et al. 2015). The antioxidant peptide has the ability to confer protective effects against mutant mitochondria and synaptic toxicities (Hemachandra Reddy et al. 2017). Hao et al. also reported that SS31 can reduce age-related activation of NFκB in mice (Hao et al. 2016). Hemachandra et al. observed SS31 was able to cross the blood brain barrier and reached the mitochondria. SS31 was found to enhance mitochondrial biogenesis and synaptic activity by reducing Aβ production, reducing mitochondrial dysfunction and maintaining mitochondrial dynamics in AD mouse models (Reddy et al. 2017).

Mitochondria-permeable antioxidants such as edaravone, idebenone, α-lipoic acid, carotenoids, vitamin E, and coenzyme Q10, and mitochondria-targeted antioxidants such as MitoQ, SkQ and astaxanthin (a ketocarotenoid from the xanthophyll family) are of increasing interest (Zhang et al. 2016). Balietti et al. reported astaxanthin as having a gender-related effect on ageing rat brain by exerting anti-inflammatory effects differentially in male and female brains (Balietti et al. 2016). Idebenone, MitoQ and SkQ have been specifically explored for the treatment of AD (Zhang et al. 2016; Selfridge et al. 2013). Idebenone (2,3-dimethoxy-5-methyl-6-(10-hydroxydecyl)-1,4-benzoquinonenoben), a short chain benzoquinone is structurally similar to coenzyme Q10 and functions both as an antioxidant and electron carrier, however, its clinical application has been limited to date because of gastrointestinal, neurotoxic or cardiotoxic side effects (Zhang et al. 2016). MitoQ a lipophilic molecule bearing a cation moiety, passes directly through the mitochondrial membrane where it accumulates and is recognised as an effective mitochondria-targeted antioxidant (Zhang et al. 2016). Another mitochondria targeted antioxidant is cationic SkQ [10-(6′-plastoquinonyl)decyltriphenyl-phosphonium)] which also accumulates in the mitochondria. Several studies have shown that SkQ protects cells from age related diseases. However, its safety and clinical usefulness need further investigations. Neither MitoQ nor SkQ are FDA-approved (Zhang et al. 2016).

### Targeting senescent cells

If senescent cells contribute to the generation of lipid peroxidation products as a consequence of altered metabolic processes, then therapeutically targeting senescent cells to reverse such metabolic abnormalities may be a viable approach for reducing their impact during ageing. No doubt many of the strategies outlined above would also be pertinent for targeting senescent cells. For example, strategies which target fatty acid oxidation might reduce their harmful impact on localised tissue. Pharmacological inhibition (Etomoxir) of carnitine palmitoyltransferase 1, the rate limiting step in mitochondrial fatty acid oxidation was shown to normalise metabolic activity, as determined by oxygen consumption in OIS fibroblasts (Quijano et al. 2012).

Alternatively, if indeed senescent cells are a major source of lipid peroxidation products that promote ageing, then one strategy may be to remove senescent cells from tissues altogether. In fact, drugs have emerged that could potentially eliminate senescent cells to improve health (see Table 2) [reviewed in (de Keizer 2017; Soto-Gamez and Demaria 2017; Schmitt 2017)]. More recently, a FOXO4 peptide was shown to specifically induce apoptosis in senescent cells and reverse some aspects of ageing (Baar et al. 2017). Nanoparticles loaded with drugs targeted towards senescent cells have also been developed (Agostini et al. 2012; Thapa et al. 2017).

**Table 2:** List of known compounds shown to specifically induce cell death in senescent cells

Compound	Mechanism of action	Cell type/tissue	Model	Induction	Reference
ABT-263	Inhibition of BCL-2 and BCL-xL	Fibroblasts, bone marrow hematopoietic stem cells, muscle stem cells	In vitro in vivo (mouse)	IR, REP, RAS	Chang et al. (2015)
ABT-737	Inhibition of BCL-W and BCL-XL	Human fibroblasts, lung, skin	In vitro in vivo (mouse)	IR, REP, ETOP, RAS, p14ARF	Yosef et al. (2016)
Dasatinib	Unknown, kinase inhibitor	Human fat cell progenitors	In vitro in vivo (mouse)	IR	Zhu et al. (2015)
Piperlongumine	Unknown, ROS-independent	Human fibroblasts	In vitro	IR, REP, RAS	Wang et al. (2016)
Quercetin	Unknown	Human endothelial cells, mouse BM-MSCs	In vitro in vivo (mouse)	IR	Zhu et al. (2015)

*IR* ionised irradiation; *REP* replicative exhaustion; *RAS* oncogenic Ras-induced, *ETOP* etoposide induced; *p14ARF* p14 overexpression (Yosef et al. 2016; Zhu et al. 2015; Wang et al. 2016; Chang et al. 2015)

Finally, rather than waiting until senescent cells are present and then tackling the problems they cause, it would be better to prevent/reduce their appearance through life-style changes. For example, it has been demonstrated in mice that exercise can prevent diet-induced cell senescence and short-term dietary restriction has been shown to reduce the number of senescent cells in tissue of mice (Schafer et al. 2016; Wang et al. 2010). It was also suggested that dietary restriction improves mitochondrial function in tissues through reduction in cell senescence.

## Conclusions

Ageing and age-related diseases have been associated with mitochondrial uncoupling and elevated ROS formation. Dysfunctional mitochondria predispose to altered lipid metabolism and increased lipid peroxidation products. Mitochondrial antioxidants that can restore function and prevent pathological lipid peroxidation are showing promise in slowing biological ageing and therefore they may offer benefit for slowing the progression to age-related diseases such as neurodegeneration. In parallel, newer drug classes are providing a different strategy to delay ageing through removal of senescent cells. Using these drugs as tools offer an opportunity to increase our understanding of whether the alterations in ROS, lipid metabolism and mitochondrial lipids observed during ageing and diseases are due to the accumulation of senescent cells.
